# Novel Antiviral and Antibacterial Durable Polyester Fabrics Printed with Selenium Nanoparticles (SeNPs)

**DOI:** 10.3390/polym14050955

**Published:** 2022-02-27

**Authors:** Tarek Abou Elmaaty, Khaled Sayed-Ahmed, Hanan Elsisi, Shaimaa M. Ramadan, Heba Sorour, Mai Magdi, Shereen A. Abdeldayem

**Affiliations:** 1 Department of Material Art, Faculty of Art & Design, Galala University, Galala 43713, Egypt; 2 Department of Textile Printing, Dyeing and Finishing, Faculty of Applied Arts, Damietta University, Damietta 34512, Egypt; hanan_gamal58@du.edu.eg (H.E.); shaimaamostafa@du.edu.eg (S.M.R.); hebasorour@du.edu.eg (H.S.); maymagdy94@du.edu.eg (M.M.); shereen_anwar@du.edu.eg (S.A.A.); 3 Department of Agricultural Chemistry, Faculty of Agriculture, Damietta University, Damietta 34512, Egypt; drkhaled_1@du.edu.eg

**Keywords:** COVID-19, protective textiles, selenium nanoparticles, printed polyester fabric, antibacterial, antiviral, cytotoxicity

## Abstract

The COVID-19 pandemic has clearly shown the importance of developing advanced protective equipment, and new antiviral fabrics for the protection and prevention of life-threatening viral diseases are needed. In this study, selenium nanoparticles (SeNPs) were combined with polyester fabrics using printing technique to obtain multifunctional properties, including combined antiviral and antibacterial activities as well as coloring. The properties of the printed polyester fabrics with SeNPs were estimated, including tensile strength and color fastness. Characterization of the SeNPs was carried out using TEM and SEM. The results of the analysis showed good uniformity and stability of the particles with sizes range from 40–60 nm and 40–80 nm for SeNPs 25 mM and 50 mM, respectively, as well as uniform coating of the SeNPs on the fabric. In addition, the SeNPs—printed polyester fabric exhibited high disinfection activity against severe acute respiratory syndrome coronavirus 2 (SARS-CoV-2) with an inhibition percentage of 87.5%. Moreover, a toxicity test of the resulting printed fabric revealed low cytotoxicity against the HFB4 cell line. In contrast, the treated fabric under study showed excellent killing potentiality against Gram-positive bacteria (*Bacillus cereus*) and Gram-negative bacteria (*Pseudomonas aeruginosa, Salmonella typhi, and Escherichia coli*). This multifunctional fabric has high potential for use in protective clothing applications by providing passive and active protection pathways.

## 1. Introduction

Viruses in the environment, as one of the pathogenic microorganisms, employ a variety of routes to infect people, posing a severe threat to public health. For example, the SARS-CoV2 (COVID-19) virus may be easily transmitted via droplets or aerosol, which then attach to the surfaces and are subsequently touched by a receiver person [1].

Fabrics are now employed in a wide range of applications and for a wide range of purposes. However, most antiviral fabrics have not been industrialized and there has been no systematic research on antiviral fabrics. As a result, antiviral textile fabrics are now in high demand owing to the requirement for hygienic and clean fabrics. Medicine, health, and hygiene are important and growing sectors of the fabric industry. Development is occurring as a result of the concurrent growth and advancement of technology in both the fabric and medicinal sectors [2]. Furthermore, antiviral agents endow the treated fabrics with antiviral properties by killing viruses on the surface of the fabric or preventing biofilm formation, thereby lowering the risk of infection and re-infection. In addition, because antiviral fabrics are reusable, they can help to reduce pollution [3], whereas the interaction method can be divided into: linking of connecting to the virus and discouragement of virus attachment penetration into the cell; generation of highly active oxygen and other ions and radicals that cohere to the wall (spikes or membrane) and destroy the structure and function of viral proteins and nucleic acids, simulating the nucleus to increase the immune response of the host cell, and inhibiting the budding and spreading of the virus [4].

Recently, metal-based nanoparticles have been found to possess unique physicochemical features due to their small size and high specific surface area, which allow them to interact with viruses and other microorganisms. Various metal and metal oxide nanoparticles (such as Ag, Cu, Ti, Au, and Zn) have been introduced as antiviral agents [5,6] because they were exhibited a wide range of antiviral activity, durability, and effectiveness at a much lower dosage [7]. In addition, they were useful in fighting viral infections. Joshi and Roy [8] created an antiviral fabric by mixed AgNO_3_ solution with different reducing agents, and then immersed the fabric in the combined solution to generate nanoparticles in situ. Gabbay et al. [9] proved that textiles impregnated with copper oxide solution have a high inactivation impact on bacteria, fungi, and HIV virus. Borkow et al. [10] impregnated polypropylene fiber with a copper oxide solution, which efficiently inactivated the influenza virus. Antiviral textiles can be made by completing antiviral materials made by attaching silver, copper, zinc, and other metals or their ions to zeolite by physical adsorption and ion exchange. Imai et al. [11] synthesized (Na_12_((AlO_2_)_12_(SiO_2_)_12_)27H_2_O) zeolite on cotton textiles to manufacture CuZeo textiles, which may be employed in the medical and health domains, using the structural components of zeolite as a raw material. Metal-organic frameworks (MOFs), as good carriers of functional NPs, can be coupled with zinc, silver, and other antiviral elements, and then impregnated or grafted onto the textile. Li et al. [12] revealed that the zinc-imidazolate (ZIF-8) MOF on textiles has a good adsorption capacity for viruses and bacteria, as well as the ability to inactivate bacteria under light. This finishing processing approach was easy to use, inexpensive, and can be used with inorganic, organic, and natural antiviral materials. However, this approach has a high initial mass release and limited durability, which reduce the comfort of the fabric. 

Selenium (Se), as an essential micronutrient, was discovered by Jons Jacob Berzilius. It belongs to the 16th group between sulphur and tellurium [13]. It existed in three well-defined allotropic forms: amorphous selenium, which is red in color; crystalline trigonal with helical chains that is black in color; and crystalline monoclinic (α, β, γ) with Se8 rings, which is also red in color. Selenium is a metalloid possessing the characteristics of both metals and a non-metal. Selenium is a photoelectrically active semiconductor where its uses include xerography, glass production, and solar cell assembly [14]. In comparison to Se compounds, elemental Se in nano size [Se nanoparticles (SeNPs)] has emerged as a new study target, since it has been discovered to have coloring capabilities [15,16,17,18] as well as antimicrobial, antitumor, antioxidant, and antibiofilm properties [19,20,21,22,23]. Moreover, SeNPs exhibited low cytotoxicity [15,24]. They have contributed to a variety of health-promoting activities and actions aimed at disease prevention and treatment [25,26] and have received considerable attention in the literature [15,16,27,28,29,30,31]. However, to the best of our knowledge, no study has been reported prior to our investigation of the utilization of SeNPs as antiviral agents for all types of fabrics. The present study proved that SeNPs as a novel antiviral agent were deposited on polyester fabric via a flat screen-printing technique, which effectively inhibited the growth of the SARS-CoV-2 virus, *Bacillus cereus, Pseudomonas aeruginosa, Salmonella typhi,* and *Escherichia coli* bacteria. Characterization of the fabric revealed that the size of the SeNPs was ~40–80 nm, with higher color strength (*K/S),* high washable resistance, and durability. Most importantly, SeNPs on the polyester fabric inhibited SARS-CoV-2 propagation in vitro by killing the virus.

## 2. Experiment 

### 2.1. Materials 

A 100% plain-weave polyester (PET) fabric with wight of 108 g/m^2^ and it was 70 denier warps and 150 denier wefts. The fabric was purchased from Omega for Textile Industries (Sharqia, Egypt). Printofix^®^ Binder N 86 (acrylate-based copolymer, anionic, SPI, Cairo, Egypt), Fast print^®^ thickener 600 (polyacrylate inverse emulsion, anionic, SPI, Delta for Chemical Industry Co., Cairo, Egypt). Polyvinylpyrrolidone (PVP), sodium biselenite, and ascorbic acid, were purchased from Loba Chemie (Mumbai, India). All chemicals were used as received.

### 2.2. Methods 

#### 2.2.1. Synthesis of SeNPs

SeNPs were synthesized through a green route according to the method described by Abou Elmaaty et al. [32], with minor modifications. Sodium biselenite was used as a precursor at varied concentrations of 50 and 100 mM. PVP was used as stabilizer to maintain the SeNPs stability and added to sodium biselenite solution at a concentration of 12 g/100. Then, ascorbic acid, as a reductant, was prepared at different concentrations of 100 mM and 200 mM to reduce sodium biselenite to SeNPs. The mixtures were mixed together at a molar ratio of 2:1 (ascorbic acid: NaHSeO_3_), and a volume ratio of 1:1. The change in solution color from colorless to orange to dark orange refers to the complete formation of SeNPs. 

#### 2.2.2. Printing Polyester Fabric with SeNPs 

The printing pastes were prepared using the recipe shown in Table 1. The paste was applied to the substrates using the flat screen-printing technique. The printed fabrics were dried at 80 °C for 3 min and cured at 200 °C for 3 min using a high-temperature steamer from (R. B Electronic and Engineering Private Limited, Valsad, Gujarat, India). Finally, the fabrics were washed with 2% non-ionic detergent at 60 °C for 10 min. More than one print paste was executed with varying curing times and temperatures with both concentrations of SeNPs (25, 50 mM) to achieve the optimum printing recipe.

### 2.3. Characterization 

#### 2.3.1. Transmission Electron Microscopy (TEM) Analysis of SeNPs

The size and morphology of the prepared SeNPs were examined using a transmission electron microscope at 200 kV (JEOL, JEM 2100F, Musashino, Akishima, Tokyo, Japan). A drop of the prepared SeNPs solution was loaded on a 400-mesh copper grid. Then, the grid was let at room temperature in the air until completely dry.

#### 2.3.2. X-ray Diffraction (XRD) Analysis

The crystalline nature of the SeNPs, the surface of polyester fabric, and the printed fabric were examined using an X-ray diffractometer (Bruker D8 ADVANCE, Karlsruhe, Germany) to confirm the formation of the prepared NPs and their sufficient deposition on the polyester surface.

#### 2.3.3. Evaluation of Color Strength (K/S) and Color Co-Ordinate Values of Polyester Fabrics

The color uptake, expressed as the color strength (*K/S*) of the blank polyester fabric and printed polyester fabrics with SeNPs, was determined using a spectrophotometer (CM3600A; Konica Minolta, Osaka, Japan). *K/S* values were evaluated at the wavelength of maximum absorption (λ_max_) of the color reflectance curve at 360 nm. The total color difference (*∆E*) is represented in terms of CIE LAB color space data. This value was calculated using (Equation (1)):

(1) $$\Delta E=\sqrt{{\left(L^{*}{}_{2}-L^{*}{}_{1}\right)}^{2}+{\left(a^{*}{}_{2}-a^{*}{}_{1}\right)}^{2}+\left(b^{*}{}_{2}-b^{*}{}_{1}\right){\;}^{2}}$$

where ΔE is the total difference between the blank polyester fabric and printed polyester fabrics with SeNPs, *L^∗^* is the lightness from black (0) to white (100), *a^∗^* is the red (+)/green (−) ratio, and *b^∗^* is the yellow (+)/blue (−) ratio.

#### 2.3.4. Color Fastness Properties of Polyester Fabrics

The color fastness of the blank polyester fabric and polyester fabrics printed with SeNPs was tested according to the following standard methods. They were determined using the AATCC (61-1996) [32], (8-1996) [33], and (16-2004) [34] tests for washing, rubbing, and light fastness, respectively. 

#### 2.3.5. Mechanical Properties of Polyester Fabrics

Tensile strength and tearing tests of blank polyester fabric and printed polyester fabrics with SeNPs were performed according to ASTM D638-14 [35] using a tensile testing machine (Zwick Z010, Ulm, Germany). Additionally, the durability of washing was evaluated according to AATCC 61(2A)-1996 [36] after five washing cycles.

#### 2.3.6. SEM and EDX of Polyester Fabrics

The morphology of the surface of both the blank fabric and the printed fabric using SeNPs, was examined by a scanning electron microscope (JEOL, Tokyo, Japan) supported by the unit of energy dispersive X-ray (EDX).

#### 2.3.7. Evaluation of the Antiviral Properties of Polyester Fabrics

The virus severe acute respiratory syndrome coronavirus 2 (SARS-CoV-2) hCoV-19/Egypt/NRC-03/2020 (Accession Number on GSAID: EPI_ISL_430820) virus (~105 PFU/mL) was added to the fabric for 60 min. This assay was performed according to the method described by Hayden et al. [37] in a six well plate where Vero E6 cells were cultivated for 24 h at 37 °C. The treated and control untreated viruses were incubated with the printed polyester fabric for 60 min. Growth medium was removed from the cell culture plates, and the cells were inoculated with (100 μL/well) virus dilution. After 1 h of contact time for virus adsorption, 3 mL of DMEM supplemented with 2% agarose was added to the cell monolayer; the plates were left to solidify and were incubated at 37 °C until the formation of viral plaques (3 to 4 days). Formalin (10%) was added for two hours, and then plates were stained with 0.1 %crystal violet in distilled water. Control wells were included where the untreated virus was incubated with Vero E6 cells and finally plaques were counted. The percentage reduction in plaque formation was compared to control wells and was recorded as follows:

% inhibition = viral count (untreated) − viral count (treated)/viral count (untreated) × 100(2)

#### 2.3.8. Evaluation of Antibacterial Properties of Polyester Fabrics

The antibacterial activity test for the printed polyester fabrics with SeNPs was conducted according to the AATCC (147-2004) test [38]. Antibacterial activity was evaluated against G+ve bacteria *(Bacillus cereus)* as well as G-ve bacteria (*Escherichia coli,* *Salmonella typhi*, and *Pseudomonas* *aeruginosa)*. The growth inhibition zone (mm) was then determined.

#### 2.3.9. Cytotoxicity Test of Polyester Fabrics

The cytotoxicity of the prepared NPs was evaluated in normal HFB4 cell lines using the MTT assay. HFB4 cells are normal human melanocytes cells, which are considered the cells most exposed to fabrics [39]. The polyester fabric under study was printed with SeNPs under optimum conditions and were sterilized and cut. Then, it was placed in a six-well tissue culture plate, inoculated with cells, and incubated at 37 °C for 24 h. The growth medium was decanted, and the cell monolayer was washed twice by washing. Physical signs of cytotoxicity were observed in the cells. The polyester fabrics printed with SeNPs were picked up, and then aliquot 20 µL of 0.5% 3-(4,5-dimethylthiazol-2-yl)-2,5-diphenyltetrazolium bromide dye (MTT) prepared in phosphate-buffered saline was mixed with each well and shaken for 5 min. The wells were incubated at 5% CO_2_ and 37 °C for 5 h. Formazan, an MTT metabolite, was formed and resuspended in 200 µL of dimethyl sulfoxide, followed by shaking for 5 min. Then, the absorbance was read at 560 nm and the background was measured at 620 nm and then subtracted.

#### 2.3.10. Statistical Analysis 

All tests have been carried out by taking the mean of three sample readings. The standard error of the mean was calculated according to the equation given below and found to be ± 0.1

(3) $$\mathrm{SEX}=\frac{S}{\sqrt{n}}$$

where *S* = sample standard deviation, and *n* = the number of observations of the sample.

## 3. Results and Discussion

### 3.1. Transmission Electron Microscopy (TEM) Analysis of SeNPs

**Figure 1:**
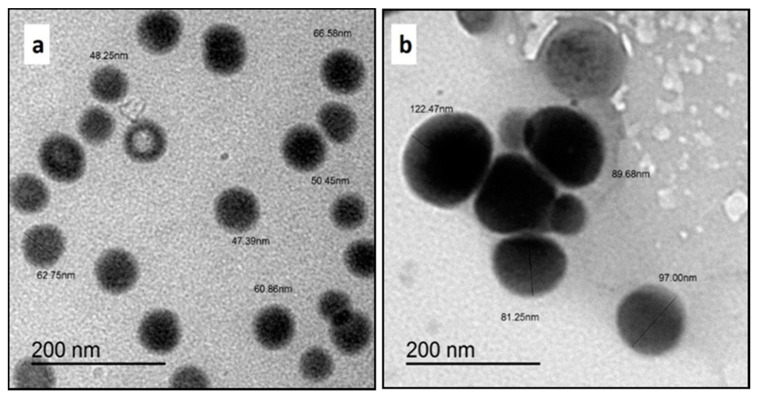
TEM micrographs of SeNPs prepared at different concentrations of (**a**) 25 mM and (**b**) 50 mM.

Transmission electron microscopy (TEM) was used to examine the morphology, shape, and size of the SeNPs produced at two different concentrations (25 and 50 mM). TEM images revealed the development of spherical SeNPs that were well-dispersed in a colloidal solution. Moreover, the obtained micrographs showed that no aggregation or deformation occurred in the colloidal SeNPs solution. Most SeNPs prepared at a low concentration (25 mM) ranged in size from 35 nm to 80 nm. In contrast, the majority of SeNPs at a high concentration of 50 mM exhibited diameters between 40 and 155 nm, demonstrating that the diameter of the SeNPs increased with the increase in NP concentration. In addition, SeNPs at a low concentration were different in shape, including spherical and ring-shaped particles, whereas SeNPs at a high concentration (50 mM) were ordinary solid spherical particles. The obtained micrographs illustrated that SeNPs at a low concentration had a higher specific surface area as a result of the decrease in size and the presence of a hollow shape in the morphology, as displayed in Figure 1.

### 3.2. XRD Analysis

**Figure 2:**
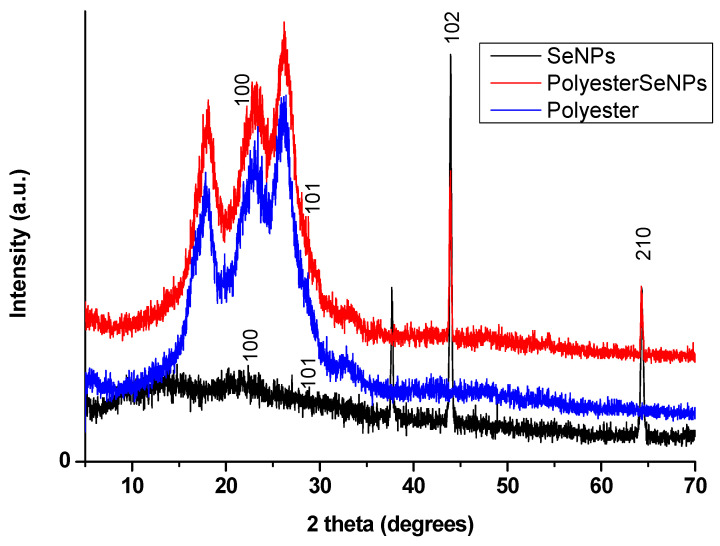
XRD patterns of the prepared SeNPs, polyester fabric, and the printed polyester fabric.

XRD analysis was conducted to confirm the formation of SeNPs and their sufficient deposition on the surface of the printed polyester fabric, depending on the crystallinity nature of SeNPs. As shown in Figure 2, the synthesized SeNPs either in the colloidal solution or that deposited on the surface of the printed polyester fabric were highly crystalline. In addition, the XRD patterns at 24.28°, 29.24°, 43.64° and 64.28° corresponded to 100, 101, 102 and 210 crystal planes, respectively, depending on the JCPDS 86-2246 international database [40].

### 3.3. Evaluation of Color Strength (K/S) and Color Co-Ordinate Values of Polyester Fabrics

**Figure 3:**
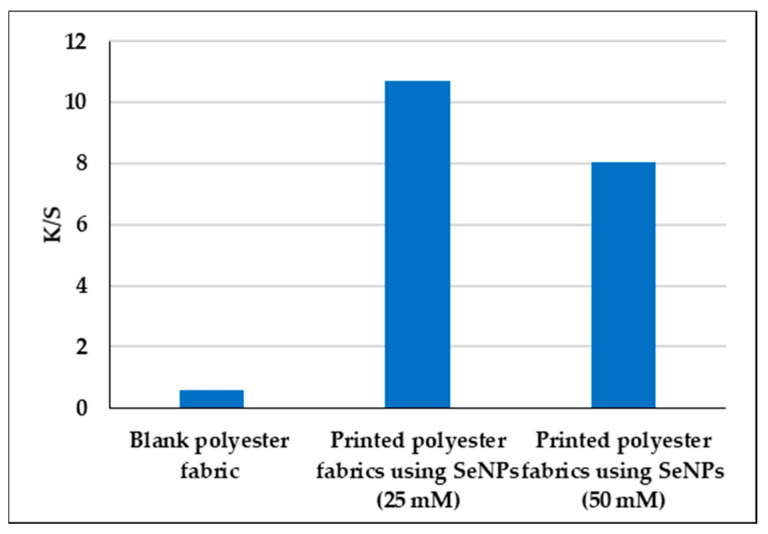
Color strength (*K/S*) of blank polyester fabric and printed polyester fabrics with SeNPs.

As shown in Figure 3, the *K/S* values of the blank polyester fabric, printed polyester fabric with SeNPs (25 mM) and (50 mM) were 0.60, 10.68 and 8.02 respectively. The maximum color strength was observed at 360 nm. The color strength decreased by 24.91% for printed polyester fabrics with SeNPs (50 mM) compared to printed polyester fabric with SeNPs (25 mM). This can be attributed to an extensive increase in the positive charges of Se^+^ ions at higher concentration of SeNPs with higher repulsion forces with each, which led to a decrease in the ionic bonding process between the polyester fabric and SeNPs. Thus, the color yield significantly increased for the polyester fabric printed with SeNPs 25 mM (as shown in Figure 3). Finally, a shade was observed with higher color strength.

The color coordinates of the blank polyester and polyester fabrics printed with SeNPs were evaluated using a spectrophotometer (CM3600A; Konica Minolta, Japan). The color coordinate values of the tested polyester fabrics are listed in Table 2. As shown in Table 2, the high (L*) value of (91.6) and negligible (a*, b*) values of (0.29) and (1.35) for the blank polyester fabric indicated the white color of the fabric. As for the *K/S* values of printed polyester fabrics of both concentrations of SeNPs, a decrease in the values of L* was also observed, which indicated an increase in the absorbance of SeNPs. From the (a*) and (b*) values of the printed polyester fabrics with SeNPs (25 mM), the color of the fabric turned red and finally appeared yellow/brownish. For printed polyester fabrics with SeNPs (50 mM), the color of the fabric was darkened and appeared a darker yellow/brownish.

### 3.4. Effect of Curing Temperature on the Color Strength (K/S) of Polyester Fabrics

**Figure 4:**
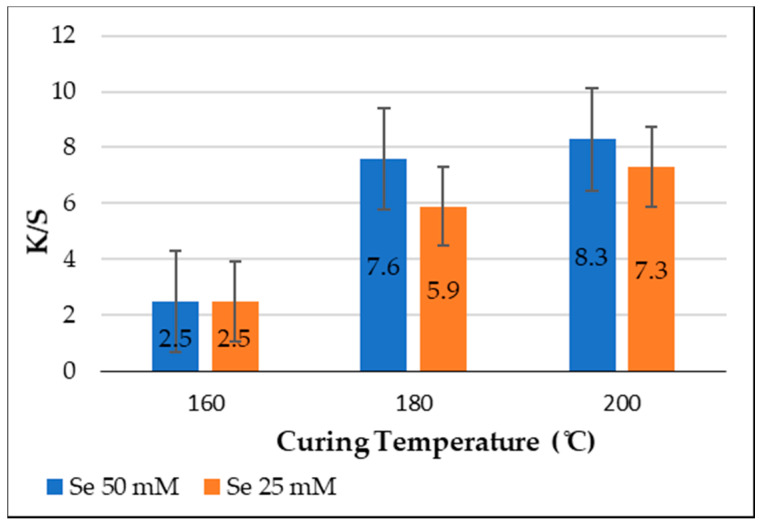
Effect of curing temperature on color strength (*K*/*S*).

For printed polyester fabrics with SeNPs at a constant time of 5 min and concentrations of 50 and 25 mM, the influence of curing temperatures from 160 to 200 °C was tested. As shown in Figure 4, the K/S values for both concentrations of SeNPs significantly improved and showed a clear increase from 160 to 200 °C. This may be attributed to the fact that increasing the temperature increased the uptake of NPs, and consequently the color strength. This increase in *K/S* values could be inferred as facilitating NP release from the thickener film, improving the swell ability of the printed fabrics and the availability of their active sites, and thus providing more opportunities for adsorption, accommodation, and fixation of NPs, resulting in a greater depth of shade [41].

### 3.5. Effect of Curing Time on Color Strength (K/S) of Polyester Fabrics

**Figure 5:**
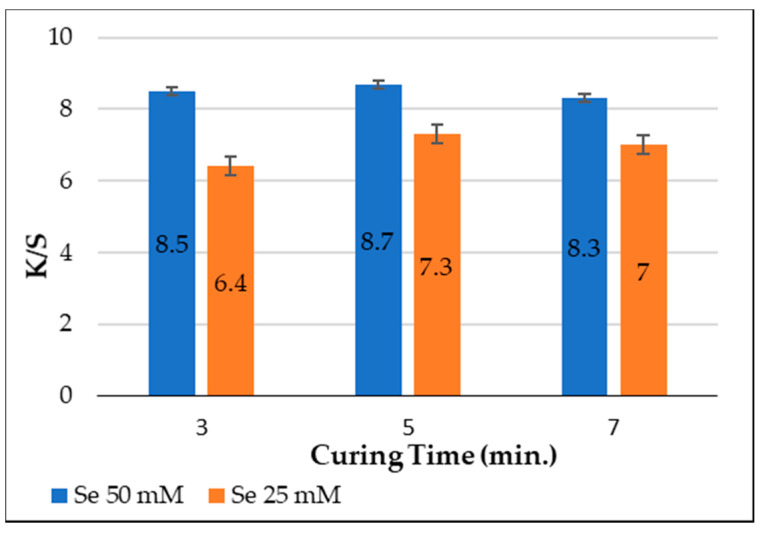
Effect of curing time on color strength (*K*/*S*).

The impact of curing time on the K/S of printed polyester fabrics with SeNPs was tested at various curing times (3, 5, and 7 min), and at a constant curing temperature of 200 °C, and SeNPs concentrations of 50 and 25 mM. Figure 5 shows that the K/S values of the fabrics under study employing both 50 and 25 mM SeNPs concentrations increased with the curing time in the 3-to-5-min range, but was somewhat decreased with further increases. This may be attributed to the fact that the binder, which enclosed the SeNP molecules, formed a film and saturated the pores of fabric under curing conditions [16]. The curing time allows SeNPs to interact with polyester molecules, enhancing the possibility of increasing the reaction efficiency. The results showed that 5 min was sufficient for efficient printing without compromising the physical and mechanical quality of the fabric.

### 3.6. Color Fastness Properties of Polyester Fabrics

The washing, rubbing, and light fastness of polyester fabrics printed with SeNPs were tested under optimum conditions. The washing and rubbing fastness were outstanding, even after five washing cycles, according to the results presented in Table 3. In addition, light fastness was found to be good to very good. The production of metal chelates may be responsible for the fixation of SeNPs onto polyester fabric [2]. 

### 3.7. Mechanical Properties of Polyester Fabrics

**Figure 6:**
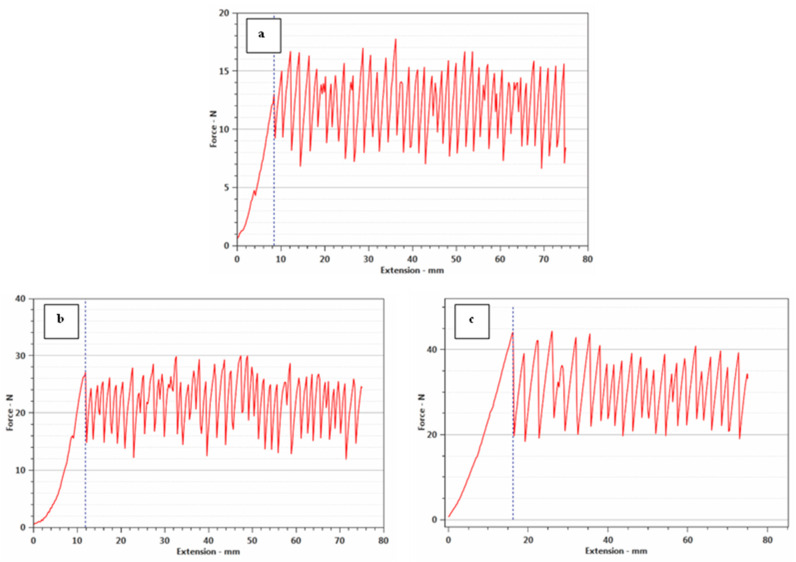
Tearing strength of (**a**) untreated polyester fabric; (**b**) printed polyester fabric with 25 mM of SeNPs; and (**c**) printed polyester fabric with 50 mM of SeNPs.

The elongation at break and maximum force at break (N) of the printed polyester fabrics with SeNPs were measured to evaluate the mechanical performance of the polyester fabrics before and after printing with SeNPs, as reported in Table 4 compared to the blank polyester, the printed fabrics demonstrated an increase in elongation and maximum force at break. The increase in strength after printing with SeNPs can be explained by the creation of a film on polyester fabrics with inter-fiber crosslinking via hydrogen bonding. As a result, the tensile force is distributed symmetrically throughout the fabrics [1]. From Figure 6, it is concluded that the printing of polyester fabric with SeNPs lead to an increase in tearing strength in both concentrations of SeNPs; this was due to the SeNP layer on the fabric’s surface and the limitation of yarn movement during tearing. When compared to blank polyester fabric, the presence of the SeNP layer minimized severe fabric degradation and assisted in the retention of fibres within the fabric matrix [42].

### 3.8. SEM and EDX Polyester Fabrics

**Figure 7:**
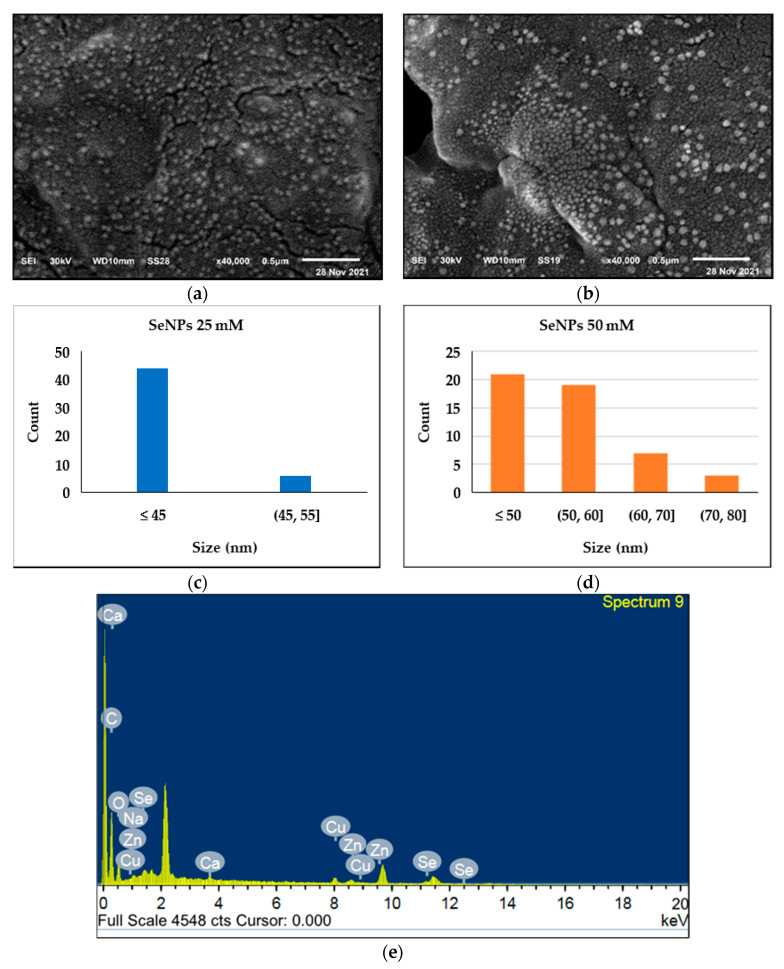
SEM micrographs of (**a**) polyester fabric printed with SeNPs (25 mM) under optimum conditions and (**b**) polyester fabric printed with SeNPs (50 mM) under optimum conditions, as well as (**c**,**d**) the size distribution histogram of the 25 mM and 50 mM SeNPs on polyester fabric; and in addition, (**e**) the EDX spectrum of the printed polyester fabric with SeNPs.

SEM micrographs of polyester fabrics printed with SeNPs at different concentrations of 25 mM and 50 mM showed that the fabric surface was sufficiently coated with an obvious layer of the synthesized SeNPs. Additionally, the SEM images showed that SeNP distribution on the polyester fabric surface was uniform and homogeneous. EDX analysis was used to determine the chemical elements present on the surface of the printed fabrics. The peaks around 1, 11 and 12.5 Kev approximately correspond to SeNPs [43]. SEM images and EDX spectrum confirmed the sufficient deposition of SeNPs on the polyester fabric, indicating the applicability of SeNPs in the printing of polyester fabrics, as shown in Figure 7. In general, the immobilization of nanometals on the surface of polymers can be performed using electrostatic interactions, covalent bonds, and ligand–receptor pairing. Various techniques exist for polyester functionalization include the active ester sites. Selenium can interact with esters by a hydrogen bond, which enhances the SeNP stability on the fabric surface [44,45]. The metal salts such as sodium, calcium and zinc salts are required during the curing of binding agents. Zinc salt is preferable in the curing of the acrylic binders. Therefore, the printing paste may be the main potential source of zinc element that illustrates the presence of Zn peak in the EDX spectrum [46,47].

### 3.9. Evaluation of the Antibacterial Properties of Polyester Fabric

The antibacterial activity of polyester fabrics printed with SeNPs at different concentrations (25 mM and 50 mM) was evaluated against various bacterial strains, including *Escherichia coli, Pseudomonas aeruginosa*, and *Salmonella typhi*, as gram-negative bacteria, and *Bacillus cereus* as gram-positive bacteria. The results listed in Table 5 illustrate that the polyester fabrics printed with the SeNP colloidal solution showed a promising antibacterial activity against the tested bacterial strains compared to the standard drugs of ciprofloxacin and tetracycline. Fabric printed with 25 mM SeNPs was more effective against all bacterial strains than fabric printed with a higher concentration of 50 mM. This variation may be due to the difference in the average sizes of the prepared SeNPs. The SeNPs prepared at a concentration of 25 mM exhibited a smaller average size than those synthesized at a higher concentration (50 mM). SeNPs at a low concentration had a higher specific area and were in contact with bacterial cells than SeNPs (50 mM) [31]. The antibacterial effect of the prepared SeNPs against the tested bacterial strains could be explained by the formation of reactive oxygen species and/or the release of ions that cause DNA damage [48]. The deposition of SeNPs on the polyester fabrics protected the fabric against the harmful effects of bacteria and made it more durable.

### 3.10. Evaluation of Antiviral Properties of Polyester Fabric

As shown in Table 6, the printed polyester fabric with SeNPs (50 mM) showed a high disinfection activity against severe acute respiratory syndrome coronavirus 2 (SARS-CoV-2) with an inhibition percentage of 87.5% after 1 h post-treatment. The printed polyester fabrics with SeNPs (25 mM) showed a medium disinfection activity, with an inhibition percentage of 25%. The antiviral activity of the printed polyester fabrics with SeNPs could be because the microbial cell walls at a neutral pH are negatively charged due to the abundance of negative carboxyl and phosphate groups rather than positive amino groups. Therefore, positively charged Se^+^ ions are tightly attached to negatively charged microbes because of electrostatic forces [49]. Thus, an increase in the concentration of SeNPs on the polyester fabric led to an increase in Se^+^ ion release, thus increasing the bond with the fabric. Owen et al. studied the stability of model human coronavirus on the different types of textiles. Coronavirus was more stable on the polyester fabric than other fabrics and remained infectious on the polyester surface for 72 hrs. The inhibition caused by the polyester fabric did not exceed 10% after 1 h [50].

### 3.11. Cytotoxicity Test

The cytotoxicity of the printed polyester fabrics with SeNPs (25 mM and 50 mM) was tested using the MTT assay against a healthy human melanocyte cell line (HFB4). The viability of the cells of the printed polyester fabric with SeNPs (25 mM and 50 mM) was 98.68% and 80.73%, respectively. However, the average relative cell viability was over 70%, as mentioned in [51], and it was concluded that the printed polyester fabrics with SeNPs have a low toxicity toward human skin.

## 4. Conclusions

In the current study, we succeeded in decorating polyester fabrics with novel antiviral agents of SeNPs to obtain multifunctional properties, including antiviral activities as well as coloring and antibacterial properties. These fabrics were fully characterized via instrumental identification, which illustrated that the particle sizes of SeNPs on polyester fabric were 40–60 nm and 40–80 nm for SeNPs 25 mM and 50 mM SeNPs, respectively. The polyester fabric was coated with nanoparticles by using a flat screen technique as a homogenous layer. In addition, after studying the effect of curing temperature, curing time, and selenium concentration, the results proved that the optimal conditions for applying the treatment were 200 °C for 5 min and 25 mM of SeNPs. Moreover, the printed polyester fabrics exhibited excellent wash and rubbing fastness, even after five washing cycles. The bactericidal properties of polyester fabrics with SeNPs were examined against *Salmonella typhi, Bacillus cereus*, *Escherichia coli,* and *Pseudomonas aeruginosa.* It demonstrated excellent antibacterial ability, specifically when printed with 25 mM SeNPs. Furthermore, the virucidal properties of polyester fabrics with SeNPs against the SARS-Cov-2 virus were also examined, and 87.5% efficiency was found. Moreover, the resulting printed fabric exhibited low cytotoxicity against the HFB4 cell line. This formulation may be used in medical applications and in manufacturing textile products for daily use to fight against COVID-19 and other bacteria.

## Figures and Tables

**Table 1:** Recipes of the printing paste.

Printing Paste Components	g/kg Paste
Thickener	20
Binder	100
SeNPs solution	880
Total	1000

**Table 2:** Color co-ordinate values of tested polyester fabrics.

Type	Sample	L*	a*	b*	C*	ΔE
Blank polyester fabric	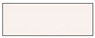	91.63	0.29	1.35	1.38	-
Printed polyester fabrics with SeNPs (25 mM)	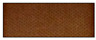	28.38	3.11	5.13	6	63.43
Printed polyester fabrics with SeNPs (50 mM)	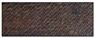	44.01	9.44	14.5	17.3	50.24

(Here, L*—Lightness, a* and b*—Parameters of color difference, C*—Saturation, Δ*E*—Color difference).

**Table 3:** Fastness properties of printed polyester fabrics with SeNPs.

Samples	Wash Fastness		Rubbing Fastness		Light Fastness	
	St.	Alt.	Dry	Wet		
Printed polyester fabrics with SeNPs (25 mM)	5	5	5	5	5	*3.5*
Printed polyester fabrics with SeNPs (50 mM)	5	5	5	5	5	*3.5*
After 5 washing cycles (Durability test)	5	5	5	5	5	*3.5*

**Table 4:** Mechanical properties of polyester fabrics printed with SeNPs.

Warp Specimen	Elongation,^a^ (%)	Maximum Force at Break,^b^ (N)
Blank polyester sample	40.66%	1069
Printed polyester fabrics with SeNPs (25 mM)	44.66%	1402.3
Printed polyester fabrics with SeNPs (50 mM)	53.34%	1340.4

^a^ Standard deviation for blank polyester = 0.8, standard deviation for printed polyester with SeNPs (25 mM) = 0.2, standard deviation for printed polyester with SeNPs (50 mM) = 0.1, ^b^ Standard deviation for blank polyester = 2.1, standard deviation for printed polyester with SeNPs = 2.1, standard deviation for printed polyester with SeNPs (50 mM) = 2.5.

**Table 5:** Inhibition zone (mm) values of polyester fabrics printed with different SeNP concentrations in addition to standard drugs.

SeNPs Concentration	Bacillus Cereus (G+)	Escherichia Coli (G-)	Pseudomonas Aeruginosa (G-)	Salmonella Typhi (G-)
Polyster fabric (blank)	-	-	-	-
Polyster printed with SeNPs (25 mM)	15	9	18	23
Polyster printed with SeNPs (25 mM) (after 5 washing cycles)	15	8	17	23
Polyster printed with SeNPs (50 mM)	12	4	16	12
Polyster printed with SeNPs (50 mM) (after 5 washing cycles)	11	3	16	12
Tetracycline (30 µg)	15	19	16	13
Ciprofloxacin (10 µg)	18	21	17	15

**Table 6:** Antiviral activity of printed polyester fabrics against Severe Acute Respiratory Syndrome Coronavirus 2 (SARS-CoV2).

Sample Code	60 min (Post-Treatment)		
	Tissue Plus Virus (PFU/mL)	Virus Control Count (PFU/mL)	Inhibition (%)
Printed polyester fabrics with SeNPs (25 mM)	6.6 × 10^5^	8.8 × 10^5^	25%
Polyster printed with SeNPs (25 mM) (after 5 washing cycles)	6.6 × 10^5^	8.8 × 10^5^	25%
Printed polyester fabrics with SeNPs (50 mM)	1.1 × 10^5^	8.8 × 10^5^	87.5%
Polyster printed with SeNPs (50 mM) (after 5 washing cycles)	1.1 × 10^5^	8.8 × 10^5^	87.5%

## Data Availability

The data presented in this study are available on request from the corresponding author.

## References

[1] Zhou J., Hu Z., Zabihi F., Chen Z., Zhu M. (2020). Progress and Perspective of Antiviral Protective Material. Adv. Fiber Mater..

[2] Masae M., Pitsuwan P., Kooptarnond K., Choopool P., Kachintararot S., Siriwan J., Ficai A. (2018). Synthesis of TiO_2_ doped selenium nanoparticles using herbal turmeric powders coating on cotton fabric for antibacterial. J. Phys. Conf. Ser..

[3] Zhang Y., Fan W., Sun Y., Chen W., Zhang Y. (2021). Application of antiviral materials in textiles: A review. Nanotechnol. Rev..

[4] Liang L., Ahamed A., Ge L., Fu X., Lisak G. (2020). Advances in Antiviral Material Development. ChemPlusChem.

[5] Bar G., Biswas D., Pati S., Chaudhary K., Bar M. (2021). Anti viral Finishing on Textiles - An Overview. Text. Leather Rev..

[6] Kampf G., Todt D., Pfaender S., Steinmann E. (2020). Persistence of coronaviruses on inanimate surfaces and their inactivation with biocidal agents. J. Hosp. Infect..

[7] Talebian S., Wallace G.G., Schroeder A., Stellacci F., Conde J. (2020). Nanotechnology-based disinfectants and sensors for SARS-CoV-2. Nat. Nanotechnol..

[8] Joshi M., Roy A. (2018). Antimicrobial textiles based on metal and metal oxide nano-particles. Nanomaterials in the Wet Processing of Textiles.

[9] Gabbay J., Borkow G., Mishal J., Magen E., Zatcoff R., Shemer-Avni Y. (2006). Copper Oxide Impregnated Textiles with Potent Biocidal Activities. J. Ind. Text..

[10] Borkow G., Zhou S.S., Page T., Gabbay J. (2010). A novel anti-influenza copper oxide containing respiratory face mask. PLoS ONE.

[11] Imai K., Ogawa H., Bui V.N., Inoue H., Fukuda J., Ohba M., Yamamoto Y., Nakamura K. (2012). Inactivation of high and low pathogenic avian influenza virus H5 subtypes by copper ions incorporated in zeolite-textile materials. Antivir. Res..

[12] Li P., Li J., Feng X., Li J., Hao Y., Zhang J., Wang H., Yin A., Zhou J., Ma X. (2019). Metal-organic frameworks with photocatalytic bactericidal activity for integrated air cleaning. Nat. Commun..

[13] Patai S., Rappoport Z. (1986). The Chemistry of Organic Selenium and Tellurium Compounds.

[14] Zhang S.-Y., Zhang J., Wang H.-Y., Chen H.-Y. (2004). Synthesis of selenium nanoparticles in the presence of polysaccharides. Mater. Lett..

[15] AbouElmaaty T., Abdeldayem S.A., Ramadan S.M., Sayed-Ahmed K., Plutino M.R. (2021). Coloration and Multi-Functionalization of Polypropylene Fabrics with Selenium Nanoparticles. Polymers.

[16] Abou Elmaaty T.M., Abdeldayem S.A., Elshafai N. (2020). Simultaneous Thermochromic Pigment Printing and Se-NP Multifunctional Finishing of Cotton Fabrics for Smart Childrenswear. Cloth. Text. Res. J..

[17] Razmkhah M., Montazer M., Bashiri Rezaie A., Rad M.M. (2021). Facile technique for wool coloration via locally forming of nano selenium photocatalyst imparting antibacterial and UV protection properties. J. Ind. Eng. Chem..

[18] Elmaaty T.M.A., Raouf S., Sayed-Ahmed K. (2020). Novel One Step Printing and Functional Finishing of Wool Fabric Using Selenium Nanoparticles. Fibers Polym..

[19] Jia X., Liu Q., Zou S., Xu X., Zhang L. (2015). Construction of selenium nanoparticles/β-glucan composites for enhancement of the antitumor activity. Carbohydr. Polym..

[20] Chen W., Li Y., Yang S., Yue L., Jiang Q., Xia W. (2015). Synthesis and antioxidant properties of chitosan and carboxymethyl chitosan-stabilized selenium nanoparticles. Carbohydr. Polym..

[21] Kong H., Yang J., Zhang Y., Fang Y., Nishinari K., Phillips G.O. (2014). Synthesis and antioxidant properties of gum arabic-stabilized selenium nanoparticles. Int. J. Biol. Macromol..

[22] Wang Q., Barnes L.-M., Maslakov K.I., Howell C.A., Illsley M.J., Dyer P., Savina I.N. (2021). In situ synthesis of silver or selenium nanoparticles on cationized cellulose fabrics for antimicrobial application. Mater. Sci. Eng. C.

[23] Shakibaie M., Forootanfar H., Golkari Y., Mohammadi-Khorsand T., Shakibaie M.R. (2015). Anti-biofilm activity of biogenic selenium nanoparticles and selenium dioxide against clinical isolates of Staphylococcus aureus, Pseudomonas aeruginosa, and Proteus mirabilis. J. Trace Elem. Med. Biol..

[24] Biswas D.P., O’Brien-Simpson N.M., Reynolds E.C., O’Connor A.J., Tran P.A. (2018). Comparative study of novel in situ decorated porous chitosan-selenium scaffolds and porous chitosan-silver scaffolds towards antimicrobial wound dressing application. J. Colloid Interface Sci..

[25] Srivastava N., Mukhopadhyay M. (2013). Biosynthesis and structural characterization of selenium nanoparticles mediated by Zooglea ramigera. Powder Technol..

[26] Yip J., Liu L., Wong K.-H., Leung P.H.M., Yuen C.-W.M., Cheung M.-C. (2014). Investigation of antifungal and antibacterial effects of fabric padded with highly stable selenium nanoparticles. J. Appl. Polym. Sci..

[27] Zhai X., Zhang C., Zhao G., Stoll S., Ren F., Leng X. (2017). Antioxidant capacities of the selenium nanoparticles stabilized by chitosan. J. Nanobiotechnol..

[28] Nastulyavichus A., Kudryashov S., Smirnov N., Saraeva I., Rudenko A., Tolordava E., Ionin A., Romanova Y., Zayarny D. (2019). Antibacterial coatings of Se and Si nanoparticles. Appl. Surf. Sci..

[29] Huang X., Chen X., Chen Q., Yu Q., Sun D., Liu J. (2016). Investigation of functional selenium nanoparticles as potent antimicrobial agents against superbugs. Acta Biomater..

[30] Abou Elmaaty T., Raouf S., Sayed-Ahmed K., Plutino M. (2022). Multifunctional Dyeing of Wool Fabrics Using Selenium Nanoparticles. Polymers.

[31] Abou Elmaaty T., Sayed-Ahmed K., Mohamed Ali R., El-Khodary K., Abdeldayem S.A. (2022). Simultaneous Sonochemical Coloration and Antibacterial Functionalization of Leather with Selenium Nanoparticles (SeNPs). Polymers.

[32] American Association of Textile Chemists and Colorists, AATTCC 61-1996 (1996). Colorfastness to Laundering: Accelerated.

[33] American Association of Textile Chemists and Colorists, AATCC Test Method 8-1996 (1996). Colorfastness to Crocking.

[34] American Association of Textile Chemists and Colorists, AATCC16-2004 (2004). Colorfastness to Light.

[35] ASTM International, ASTM D638-14 (2015). Standard Test Method for Tensile Properties of Plastics.

[36] American Association of Textile Chemists and Colorists, AATCC 61(2A) (1996). Colorfastness to Laundering.

[37] Hayden F.G., Cote K.M., Douglas R.G. (1980). Plaque inhibition assay for drug susceptibility testing of influenza viruses. Antimicrob. Agents Chemother..

[38] American Association of Textile Chemists and Colorists, AATTCC 147-2004 (2010). Antibacterial Activity Assessment of Textile Materials.

[39] Mosmann T. (1983). Rapid colorimetric assay for cellular growth and survival: Application to proliferation and cytotoxicity assays. J. Immunol. Methods.

[40] Vieira A., Stein E., Andreguetti D., Cebrián-Torrejón G., Domènech A., Colepicolo P., Da Costa Ferreira A.M. (2017). Sweet Chemistry: A Green Way for Obtaining Selenium Nanoparticles Active against Cancer Cells. J. Braz. Chem. Soc..

[41] Ibrahim N.A., El-Zairy E.M.R., El-Zairy M.R., Khalil H.M. (2012). Enhancing disperse printing and ultraviolet protecting of polyester-containing fabrics via pretreatment with chitosan/polyethylene glycol/dimethylol dihydroxyethylene urea. J. Ind. Text..

[42] Ahmad M.R., Ahmad N.A., Suhaimi S.A., Bakar N.A.A., Ahmad W.Y.W., Salleh J.M. Tensile and tearing strength of uncoated and natural rubber latex coated high strength woven fabrics. Proceedings of the 2012 IEEE Symposium on Humanities, Science and Engineering Research.

[43] Sharma G., Sharma A., Bhavesh R., Park J., Ganbold B., Nam J.-S., Lee S.-S. (2014). Biomolecule-Mediated Synthesis of Selenium Nanoparticles using Dried Vitis vinifera (Raisin) Extract. Molecules.

[44] Makvandi P., Iftekhar S., Pizzetti F., Zarepour A., Zare E.N., Ashrafizadeh M., Agarwal T., Padil V.V.T., Mohammadinejad R., Sillanpaa M. (2021). Functionalization of polymers and nanomaterials for water treatment, food packaging, textile and biomedical applications: A review. Environ. Chem. Lett..

[45] Ferro C., Florindo H.F., Santos H.A. (2021). Selenium Nanoparticles for Biomedical Applications: From Development and Characterization to Therapeutics. Adv. Healthc. Mater..

[46] Machotová J., Kalendová A., Zlámaná B., Šňupárek J., Palarčík J., Svoboda R. (2020). Waterborne coating binders based on self-crosslinking acrylic latex with embedded inorganic nanoparticles: A comparison of nanostructured ZnO and MgO as crosslink density enhancing agents. Coatings.

[47] Rattee I.D. (1997). Improvements in Fabric Printing. European Patent Office. Patent No. 97301520.9. https://patents.google.com/patent/EP0799930A2/en.

[48] Xiang J., Ma L., Su H., Xiong J., Li K., Xia Q., Liu G. (2018). Layer-by-layer assembly of antibacterial composite coating for leather with cross-link enhanced durability against laundry and abrasion. Appl. Surf. Sci..

[49] Ahmed T., Ogulata R.T., Sezgin Bozok S. (2021). Silver nanoparticles against SARS-CoV-2 and its potential application in medical protective clothing—A review. J. Text. Inst..

[50] Owen L., Shivkumar M., Laird K. (2021). The Stability of Model Human Coronaviruses on Textiles in the Environment and during Health Care Laundering. mSphere.

[51] Kangwansupamonkon W., Lauruengtana V., Surassmo S., Ruktanonchai U. (2009). Antibacterial effect of apatite-coated titanium dioxide for textiles applications. Nanomed. Nanotechnol. Biol. Med..

